# A Rare Treat

**Published:** 1905-10

**Authors:** 


### A RARE TREAT.

### Go to Dallas October 30 and See a Master Operate and Hear Him Talk.

The medical profession of Texas will have a grand rally at Dallas, October 30, on the occasion of Dr. Joseph Price's visit to that city as the guest of the Dallas County Medical Society. Drs. J. B. Deavers and Lewis S. McMurtry, both masters in their specialties, are also expected, but at this writing it is not definitely settled. The physicians of Texas must turn out and show their appreciation of the honor conferred upon them by the visit of these world celebrities. I am advised that the social program arranged for the occasion will consist of a swell reception and ball, a banquet with toasts and responses; all of which will be the swellest ever known in the history of Texas. There will also be a surgical clinic at the hospital. Doctor, don't miss it. It will be a delight and highly educational. Go. The program has not been received to date of going to press, but it is known that speeches will be made by representative men in response to toasts, and that there will be "a sound of revelry," and the bright eyes of our Texas beauties will furnish the inspiration in large quantities.

